# Brain activity associated with quadriceps strength deficits after anterior cruciate ligament reconstruction

**DOI:** 10.1038/s41598-023-34260-2

**Published:** 2023-05-17

**Authors:** Cody R. Criss, Adam S. Lepley, James A. Onate, Brian C. Clark, Janet E. Simon, Christopher R. France, Dustin R. Grooms

**Affiliations:** 1 Translational Biomedical Sciences, Graduate College, Ohio University, Athens, OH USA; 2 Ohio Musculoskeletal and Neurological Institute (OMNI), Grover Center W283, 1, Ohio University, Athens, OH 45701-2979 USA; 3 School of Kinesiology; Exercise and Sport Science Initiative, University of Michigan, Ann Arbor, MI USA; 4 School of Health and Rehabilitation Sciences, The Ohio State University, Columbus, OH USA; 5 Department of Biomedical Sciences, Ohio University, Athens, OH USA; 6 Division of Athletic Training, School of Applied Health Sciences and Wellness, College of Health Sciences and Professions, Ohio University, Athens, OH USA; 7 Department of Psychology, College of Arts and Sciences, Ohio University, Athens, OH USA; 8 Division of Physical Therapy, School of Rehabilitation and Communication Sciences, College of Health Sciences and Professions, Ohio University, Athens, OH USA

**Keywords:** Orthopaedics, Motor control

## Abstract

Prolonged treatment resistant quadriceps weakness after anterior cruciate ligament reconstruction (ACL-R) contributes to re-injury risk, poor patient outcomes, and earlier development of osteoarthritis. The origin of post-injury weakness is in part neurological in nature, but it is unknown whether regional brain activity is related to clinical metrics of quadriceps weakness. Thus, the purpose of this investigation was to better understand the neural contributions to quadriceps weakness after injury by evaluating the relationship between brain activity for a quadriceps-dominated knee task (repeated cycles of unilateral knee flexion/extension from 45° to 0°), , and strength asymmetry in individuals returned to activity after ACL-R. Forty-four participants were recruited (22 with unilateral ACL reconstruction; 22 controls) and peak isokinetic knee extensor torque was assessed at 60°/s to calculate quadriceps limb symmetry index (Q-LSI, ratio of involved/uninvolved limb). Correlations were used to determine the relationship of mean % signal change within key sensorimotor brain regions and Q-LSI. Brain activity was also evaluated group wise based on clinical recommendations for strength (Q-LSI < 90%, n = 12; Q-LSI ≥ 90%, n = 10; controls, all n = 22 Q-LSI ≥ 90%). Lower Q-LSI was related to increased activity in the contralateral premotor cortex and lingual gyrus (*p* < .05). Those who did not meet clinical recommendations for strength demonstrated greater lingual gyrus activity compared to those who met clinical recommendations Q-LSI ≥ 90 and healthy controls (*p* < 0.05). Asymmetrically weak ACL-R patients displayed greater cortical activity than patients with no underlying asymmetry and healthy controls.

## Introduction

Anterior cruciate ligament (ACL) rupture is a common knee injury in athletes, military, and other active populations, typically a result of excessive knee torsional or rotatory force during pivoting or cutting^1^. Many patients with ACL injuries, and especially athletes who desire to return to sport, undergo surgical reconstruction (ACL-R) to restore knee mechanical stability^2^. Despite surgical intervention and comprehensive rehabilitation 14–23% of patients exhibit persistent quadriceps muscle weakness upon returning to sport^3^. Unfortunately, prolonged quadriceps weakness after injury can cause gait disturbances^4^, functional impairment^5^, increased risk for reinjury, and the development of post-traumatic osteoarthritis^6,7^.

Many investigations have speculated neurological contributors to muscle weakness, which can be a result of the disruption from any one of several components within the neuromuscular system^8–10^. These components include volitional muscle control, motor unit recruitment, descending cortical drive, muscle synergies, spinal reflexes and cortical reorganization^11–18^. Lepley et al.^11^ and Zarzycki et al.^12^ reported a relationship between changes in corticospinal excitability to elicit a quadriceps muscle contraction to be linked with persistent weakness after ACL injury. These initial investigations also measured changes in the excitability of the quadriceps’ Ia afferent spinal reflex pathway, but reported conflicting results, likely related to the variable time since injury of the reports. As quadriceps strength deficits further from injury may be more dependent on a cortical responses as spinal inhibition decreases with time^14^. The use of single and paired-pulse transcranial magnetic stimulation has yielded insight into inhibitory and excitatory intracortical circuitry changes in the motor cortex of ACL-R patients, with previous reports showing increased motor thresholds (e.g. reduced corticospinal excitability) and intracortical inhibition for an evoked quadriceps contraction within the injured limb^14,15^. These measures appear to be related to strength following ACL-R, where greater levels of intracortical inhibition is associated with reduced voluntary activation and quadriceps strength^13,15^. Collectively, these studies suggest that following ACL injury, the neuromuscular system may be compromised at various levels of the nervous system.

Cross-sectional examination of ACL-R patients relative to healthy adults using neuroimaging have indicated potential widespread cortical and cerebellar reorganization (i.e. primary and secondary sensorimotor areas, cerebellum and the lingual gyrus) for knee movement^16,19,20^. However, inferences made by these comparisons have been limited to group differences, and it remains unclear whether these neurological observations are related to clinical measures of quadriceps strength. Therefore, our purpose was to determine whether regional brain activity during a quadriceps-dominated volitional knee task and clinical measures of strength asymmetry are related in individuals with ACL injury. Building on prior studies which conducted whole brain analyses, we completed an a priori ROI analysis using regions within the sensorimotor system found to be task active and demonstrated in prior literature to be uniquely different between healthy participants and individuals after ACL injury^19^. Further, as a secondary aim, we compared brain activity in regions related to strength asymmetry among ACL-R patients who did and did not meet clinical recommendations and healthy adults.

## Methods

### Patient recruitment

This investigation is a secondary data analysis on a prior published study on the same cohort that examined brain activity of the same selected ROIs related to patient-reported outcomes^21^. The following investigation occurred at two different sites (Ohio State University Center for Cognitive and Behavioral Brain Imaging and the University of Connecticut Brain Imaging Research Center) to increase sample size and generalizability and employed the same methodology (demographical survey data, fMRI motor paradigm, quadriceps strength measurements, inclusion/exclusion criteria) and very similar imaging parameters (supplemental materials). Participants between the ages of 16–35 with a history of primary, unilateral ACL-R were recruited from local physician and orthopedic offices. Both left and right unilateral ACL-R participants were included. Exclusion criteria included history of previous knee injury (e.g. meniscal injury, ligamentous tears, fractures), concussion or head injury in the past 6 months, neurologic impairment, migraines, currently taking neurologically active medications, and intracranial metallic clips. Participants at both locations were provided written and informed consent, with all procedures approved by the Universities’ Institutional Review Boards (Ohio State University and University of Connecticut) for all study activities and all methods were performed in accordance with the required institutional review board guidelines and regulations. For both primary and secondary aims of the study, a total of twenty-two participants with ACL-R were recruited (8 males/14 females; Age 22.1 ± 2.6 years; BMI 23.7 ± 3.2; involved isokinetic strength (60°/s) 2.3 ± 0.55 Nm/Kg; Tegner 7.5 ± 1.3; injured limb, 15 left/7 right; time from surgery 4.6 ± 2.6 years, 21 right leg dominant and 1 left leg dominant). Leg dominance was determined by what leg the participants would prefer to kick a ball with^22^.

Further, for the secondary aim of this investigation, twenty-two (8 males/14 females) healthy matched controls, Age 22.9 ± 2.7 years; BMI 22.8 ± 2.4; , 21 right leg dominant and 1 left leg dominant; matched limb isokinetic strength (60°/s) 2.2 ± 0.68 Nm/Kg; Tegner 8 ± 1.6) were enrolled. All participants were active and currently or previously engaged in sport (e.g., basketball, football soccer, martial arts, and hockey). To maintain the same number of left and right motor tasks among the ACL-R and control groups, control participants completed the fMRI paradigm using the same limb as the matched ACL-R limb.

### Isokinetic strength testing

Quadriceps strength was assessed using isokinetic maximal voluntary contractions (MVC). Participants were secured into an isokinetic dynamometer using both shoulder and lap straps (Biodex Medical Systems 4, Shirley, New York, USA) with their hips and testing knee secured at 90° of flexion. During testing, participants were instructed to cross their arms over their chest. Participants performed a standardized series of three submaximal warm up trials, followed by three isokinetic MVC trials (60°/second) with verbal and visual feedback to encourage maximal effort. Maximal torque produced during the three testing trials was averaged for analysis. Measures of relative muscle weakness was calculated using the quadriceps limb symmetry index (Q-LSI) as the ratio of maximal torque of the involved (injured) limb to the uninvolved (uninjured) limb (i.e., involved isokinetic quadriceps torque/uninvolved isokinetic quadriceps torque). The “involved” limb for the control group was the same limb matched to the ACL-R participant limb.

### MRI data acquisition and motor task

Prior to each participant fMRI motor task, participants were asked to lie supine while an anatomical 3-dimensional high-resolution T1-weighted image was collected for anatomical registration. Details on the imaging parameters, data processing and paradigm can be found in the supplementary material. During the function run participants completed four blocks of unilateral knee 45**°** range of motion extension–flexion for the involved limb in the ACL patients and matched side for healthy controls. Similar movement paradigms have been implemented successfully with knee orthopedic patients, stroke survivors and healthy athletes and non-athletes^16,19,23–26^. Each participant was fitted with MRI-compatible headphones, which was used to provide auditory feedback in order to pace the execution of each knee extension or flexion via an auditory metronome at 1.2 Hz for 30 s and 30 s of rest. Participants practiced the fMRI motor task, were instructed how to complete the task during a practice session using a mock MRI, and then practiced the movement timing again just prior to the functional run in the scanner. During each practice and testing session, participants were also monitored to ensure the task was completed at 45**°** range of motion and that each cycle of flexion/extension was consistently paced.

### Whole brain task activity

The subject level task activity processing and MRI paradigm details are described in the supplemental materials. All forty-four participants (ACL-R and controls) task activity was averaged using a one-sample t-test with a z-threshold > 3.1 and *p* < 0.05 FLAME 1 + 2 mixed effects model to serve as the task activation map for further ROI generation and refinement^27–30^. As prior publications have already described ACL-R relative to control participants whole brain activity differences and this dataset includes left and right involved leg movements a whole brain contrast between groups was not examined. The results of the whole brain analysis are reported in the supplemental materials.

### Region of interest generation and relationship between neural activity and Q-LSI

To extend prior work that completed whole brain analyses between those with ACL-R and healthy matched controls^19,20,23^ we utilized an ROI approach. The ROI approach allows for an expanded sample size as those with left or right-side ACL-R can be included in one analysis. As a whole brain neural correlate analysis would be confounded by which side is moving and unique laterality effects^24^ as well as examination of voxels unlikely to be affected by history of ACL-R.

ROIs were selected from regions within the sensorimotor network found in prior whole brain analyses to respond to this specific knee fMRI motor task and demonstrate altered brain activity in those with ACL-R^19^, which included primary motor, primary somatosensory, premotor, and secondary somatosensory cortices, and the cerebellum. The lingual gyrus, a region located within the extrastriate cortex, was also an ROI due to reports indicating it as a unique region with higher levels of activity following ACL-R relative to controls related to sensory reweighting and cross-modal (proprioception-vision) processing changes associated with the lost ligament and compensations during recovery^19,20^. The bilateral lingual gyrus was selected as an ROI using the Juelich anatomical atlas (across hemispheres), located within the extrastriate cortical region of the occipital cortex. Prior work in individuals after ACL-R demonstrate increased levels of bilateral lingual gyrus activity compared to injured controls^19^. Other regions typically associated with motor control such as the basal ganglia were not included as the task did not elicit sufficient activity in the whole group average analyses (supplemental materials). Only regions selected a priori and found to be different between ACL-R and controls^19^ were included in the main result, however as the parietal cortex has unique involvement in sensory integration for motor control it was included as a post-hoc exploratory result in the supplemental materials^31,32^. The parietal cortex was split into contralateral and ipsilateral inferior and superior parietal cortices and ROI generation complete the same as the other ROIs.

To generate the ROIs the whole brain task activity z-threshold maps were multiplied by the selected anatomical regions of the Juelich Histological Atlas (binarized at a probability cutoff threshold of > 30%). This ensured each ROI included only task active voxels and were within each anatomically defined ROI region. Any overlapping voxels among ROIs were assigned to the ROI with the highest probability within the Juelich atlas. The ROI size was thus dependent on task activity, brain morphology, and anatomical region. Percent signal change (BOLD signal difference between movement and rest periods) was calculated for each ROI binarized mask using FSL featquery following the prior guidelines outlined by Mumfort et al.^33^.

Pearson product-moment correlations were used to determine the relationship of mean % signal change within each ROI and LSI scores with a prior threshold of *p *< 0.05. Partial correlations were also calculated for the same analyses but to control for any confounding effect of sex. Time from surgery was not considered as a covariate, as it was not related to Q-LSI (*r* = −0.01, *p *> 0.05). False-discovery rate correction using the Benjamini–Hochberg procedure was applied to adjust for multiple-comparisons^34^.

### Comparison of neural activity between clinical categorizes of Q-LSI

Results from a survey of international sports medicine professionals identified good quadriceps strength, operationally defined by a score of 90% quadriceps limb symmetry index (or 10% deficit of the involved limb) as 1 of the 6 important measures of a successful outcome after ACL-R^35^. Further, a side-to-side difference in peak quadriceps force output of more than 10% following ACL injury may reflect significant differences in muscle performance with a low chance of measurement error^36^. Finally, Q-LSI of 90% is a recommended criterion for determination of safe to return to sport^37,38^. Therefore, as secondary purpose of the investigation, a subgroup analysis was conducted comparing ACL-R participants who did not meet clinical recommendations for return to sport based on Q-LSI (Q-LSI < 90%), ACL-R participants who met strength symmetry cut-off recommendations (Q-LSI ≥ 90%), and healthy controls. We operationally defined those with Q-LSI < 90% as “relative muscle weakness” and those who met clinical recommendations as participants without underlying weakness^35^. Analysis of covariance (ANCOVA) with sex as a covariate was used to determine whether % signal change within ROIs differed between the three groups. The Benjamini–Hochberg procedure was used as a false-discovery rate correction and all comparisons are reported with corrected *p*-values. Effects sizes were calculated for each comparison and interpreted as small (0–0.39), moderate (0.4–0.69), and large (0.7 or greater)^39^.

## Results

### Whole brain task activity analysis

**Figure 1:**
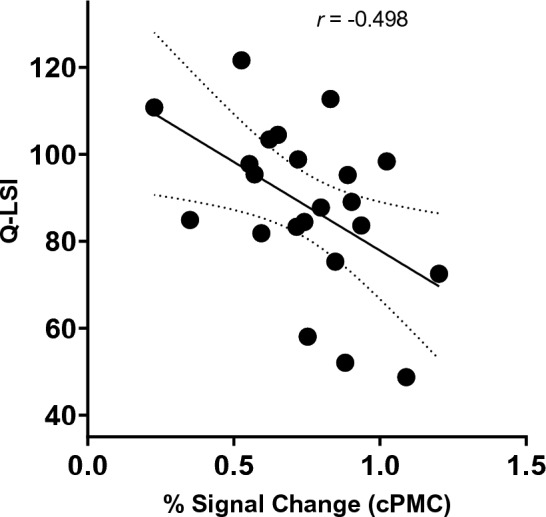
Bivariate scatterplot of % signal change of the contralateral premotor cortex and Q-LSI (quadriceps limb symmetry index) values for the ACL-R cohort.

The descriptive whole brain analyses for the task activity for all participants is described in Supplementary Table 2 and Fig. 1. The resulting activity largely confirms prior reports of a broad sensorimotor network activity for lower extremity movement. Of note this average includes left and right side knee movements as the cohort was diverse among left and right ACL-R knees, thus, the group average is for combined left and right sided movement.

### The relationship between neural activity and LSI scores in ACL-R participants

**Figure 2:**
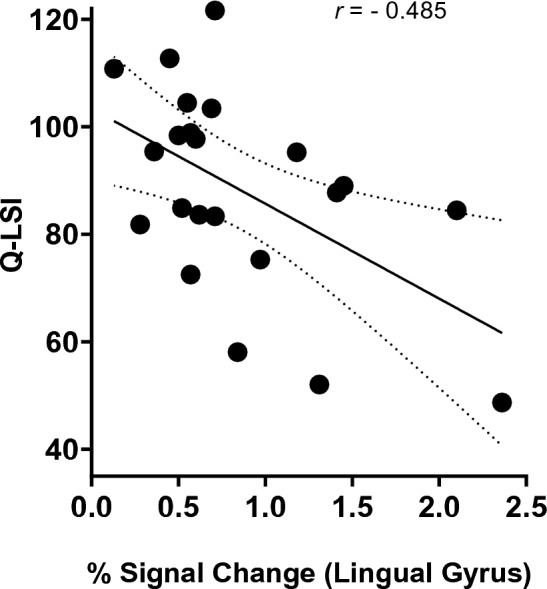
Bivariate scatterplot of % signal change of the lingual gyrus and Q-LSI (quadriceps limb symmetry index) values for the ACL-R cohort.

All correlations for the ACL-R (Table 1) and control (Table 2) groups are reported by region and correlation model (bivariate and partial for sex). There was an inverse relationship between strength asymmetry (relative weakness) and contralateral premotor cortex (PMC, *r* = −0.498,* p* = 0.02; Fig. 1), lingual gyrus (*r* = −0.485,* p* = 0.02; Fig. 2) and contralateral superior parietal lobule (*r* = −0.530 *p *= 0.011, refer to supplementary materials) activity in those after ACL-R. Partial correlations, controlling for sex exhibited similar significant relationships among neural activity within the contralateral premotor cortex (*r* = −0.499,* p* = 0.02), lingual gyrus (*r* = −0.535, *p* = 0.01), and contralateral superior parietal lobule (*r* = −0.552 * p *= 0.009; refer to supplementary materials). None of the other brain regions examined were significantly related to Q-LSI (*p* > 0.05). All correlations are provided in Table 1 for ACL-R and Table 2 for controls with Benjamini–Hochberg correction for multiple comparisons and with the raw and sex covariate r-values provided.

**Table 1:** Relationship between Q-LSI (quadriceps limb symmetry index) and ROI (region of interest) neural activity of ACL-R (anterior cruciate ligament reconstructed). Data presented as r-value and (p-value Benjamini–Hochberg corrected).

ROI	Q-LSI	Q-LSI covariates: sex
Ipsilateral M1	− 0.216 (0.33)	− 0.359(0.10)
Contralateral M1	− 0.347(0.114)	− 0.220 (0.34)
Ipsilateral S1	− 0.365 (0.10)	− 0.295(0.19)
Contralateral S1	− 0.288(0.19)	− 0.382(0.09)
Ipsilateral PMC	− 0.128 (0.57)	− 0.128 (0.57)
Contralateral PMC	** − 0.498 (0.02)**	** − 0.499 (0.02)**
Ipsilateral SMA	− 0.149 (0.509)	− 0.166 (0.47)
Contralateral SMA	− 0.159 (0.48)	− 0.149 (0.52)
Ipsilateral SII	− 0.415 (0.06)	− 0.423 (0.06)
Contralateral SII	− 0.170 (0.449)	− 0.199 (0.387)
Ipsilateral cerebellum	− 0.287(0.179)	− 0.301(0.19)
Contralateral cerebellum	− 0.398 (0.07)	− 0.398 (0.06)
Lingual gyrus	** − 0.485 (0.02)**	** − 0.535 (0.01)**

Significant values are in bold.

**Table 2:** Relationship between Q-LSI (quadriceps limb symmetry index) and ROI (region of interest) neural activity of ACL-R (anterior cruciate ligament reconstructed). Data presented as r-value and (p-value Benjamini–Hochberg corrected).

ROI	Q-LSI (controls)	Q-LSI covariates: sex
Ipsilateral M1	− 0.213(0.34)	− 0.219(0.34)
Contralateral M1	− 0.206(0.36)	− 0.207(0.37)
Ipsilateral S1	− 0.306(0.17)	− 0.316(0.16)
Contralateral S1	− 0.179(0.43)	− 0.184(0.42)
Ipsilateral PMC	− 0.215(0.34)	− 0.179(0.34)
Contralateral PMC	− 0.274(0.22)	− 0.276(0.23)
Ipsilateral SMA	− 0.233(0.30)	− 0.239(0.30)
Contralateral SMA	− 0.122(0.59)	− 0.127(0.58)
Ipsilateral SII	− 0.078(0.73)	− 0.08(0.72)
Contralateral SII	− 0.006(0.98)	− 0.004(0.99)
Ipsilateral Cerebellum	− 0.233(0.30)	− 0.256(0.26)
Contralateral Cerebellum	− 0.222(0.32)	− 0.227(0.32)
Lingual Gyrus	− 0.238(0.29)	− 0.255(0.27)

### Regional neural activity between asymmetric and symmetric ACL-R and controls

**Figure 3:**
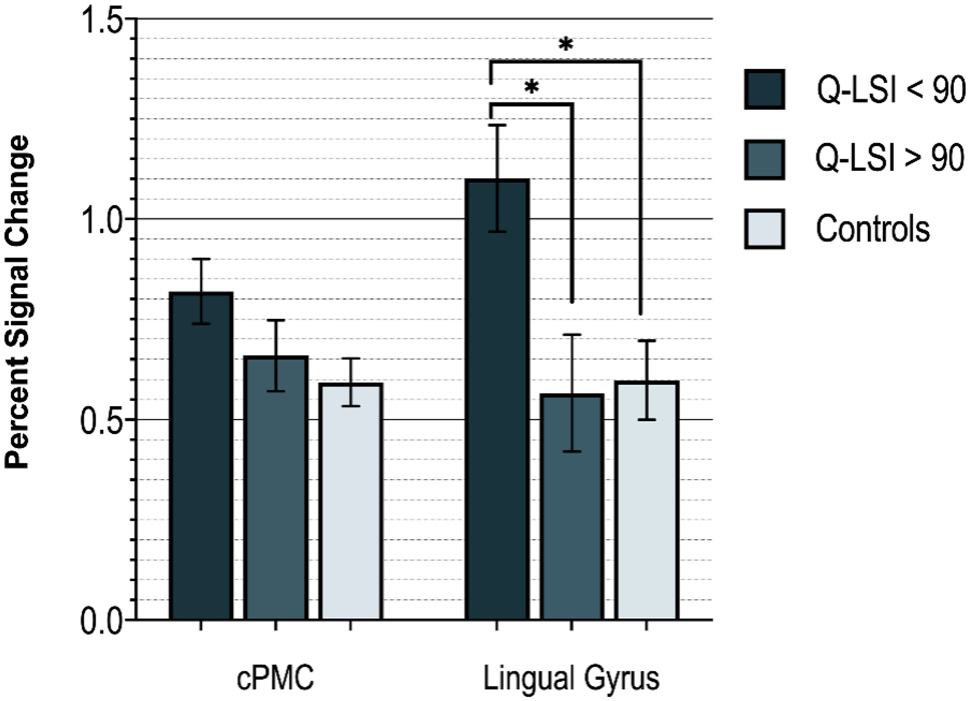
Group comparison of estimated marginal means of regional % signal change for ROIs related to strength asymmetry (covariate: sex). Error bars, standard error of the mean. Q-LSI < 90%: 12 ACL-R participants with Q-LSI < 90%; 10 ACL-R participants with Q-LSI ≥ 90%; Controls: 22 healthy participants. * indicates p-values < 0.05.

The one-way ANCOVA for the lingual gyrus was significant (F_(2,42)_ = 5.464, *p *< 0.01 η^2^ = 0.215). ACL-R participants with Q-LSI < 90% had greater levels of lingual gyrus activity compared to those who met clinical recommendation Q-LSI cut-off (mean difference 0.535 ± 0.30, *p* = 0.02) and controls (mean difference 0.503 ± 0.2, *p *= 0.01) (Fig. 3). There were no differences between ACL-R participants without strength asymmetry and healthy controls (p > 0.05).

The one-way ANCOVA for the contralateral PMC % signal change among groups was not significant (F_(2,42)_ = 2.567, *p *= 0.089). However, it should be noted that a modest effect size (η^2^ = 0.114) was observed as the ACL-R participants with Q-LSI < 90% displayed notably larger mean differences in premotor cortex activity compared to those who met clinical recommendations for Q-LSI (0.32 ± 0.30 vs. 0.161 ± 0.12). The one-way ANCOVA for the contralateral superior parietal cortex % signal change among groups was not significant (F_(2,42)_ = 1.702, *p *= 0.182, η^2^ = 0.113, supplemental materials).

### Strength data for each limb and symmetry index

Raw strength values for peak isokinetic quadriceps torque (normalized to body-weight) were calculated for each group (Q-LSI < 90%, Q-LSI ≥ 90%, healthy controls) and are provided in Table 3. Average isokinetic strength values were not different across groups for either the involved or uninvolved limb (*p* > 0.05), with average values for the involved limb being 1.96 ± 0.56 Nm/Kg, for participants with Q-LSI < 90%, 2.21 ± 0.52 Nm/Kg for participants with Q-LSI ≥ 90%, and 2.22 ± 0.68 Nm/Kg for healthy controls. Q-LSI was significantly different between groups with the Q-LSI < 90% group having 75 ± 14% Q-LSI and the Q-LSI ≥ 90% having 1.03 ± 9% Q-LSI (Table 3).

**Table 3:** Raw Isokinetic strength values (normalized to bodyweight). Isokinetic strength values for ACL-R (anterior cruciate ligament reconstruction) participants with Q-LSI (quadriceps limb symmetry index) < .90, ACL-R participants with Q-LSI ≥ .90 and healthy controls. The p-value indicates significance for the group comparison between those that achieve relative quadricep strength symmetry and those that do not, and the effect size indicates the strength of this difference. interpreted as small (0–0.39), moderate (0.4–0.69), and large (0.7 or greater).

Group	Q-LSI < 90% (n = 12)	Q-LSI ≥ 90% (n = 10)	Controls (n = 22)	P-value	Effect size
Involved isokinetic (60/s)/BW	1.96 ± 0.56	2.21 ± 0.52	2.22 ± 0.68	0.468	0.04
Uninvolved isokinetic (60/s)/BW	2.34 ± 0.65	2.51 ± 0.36	2.15 ± 0.46	0.165	0.08
Q-LSI	0.75 ± 0.14	1.03 ± 0.09	1.02 ± 0.21	< 0.001	0.372

## Discussion

### Cortical contributions to muscle function after injury

Voluntary muscle contraction requires distributed activity among several brain structures to encode movement related parameters. For example, neurons within the sensorimotor cortex regulate movement amplitude^40^ and direction^41^. Cramer et al.^42^ demonstrated that neuronal activity within the motor cortex and surrounding regions exhibit a proportional response to force output in healthy adults (more force output equated with more neural activity). Considering the peripheral neuromuscular consequences common to injury, such as joint deafferentation, reflexive inhibition, and limb disuse^43–45^, cortical reorganization is likely to occur following ACL-R. A series of recent investigations^46–48^, suggest properties of neurons along pathways projecting to the quadriceps (e.g. reductions in corticospinal excitability) are related to weakness after ACL-R. In the present study, lower levels of Q-LSI were associated with greater neuronal activity in the premotor cortex and lingual gyrus (Figs. 1 and 2 correlation and Fig. 3 by group asymmetry classification) reflecting relative increased neural demands to elicit quadriceps contractions.

### The lingual gyrus and strength asymmetry

Activity within two cortical regions (lingual gyrus and PMC) were inversely related to Q-LSI (Figs. 1 and 2). Prior case–control fMRI investigations using similar knee flexion/extension paradigms have demonstrated greater neuronal activity within the lingual gyrus in those with ACL-R compared to healthy matched controls^19,20^. Further, participants who did not meet cut-off recommendations (Q-LSI < 90%) had greater levels of lingual gyrus activity for repetitive knee flexion/extension compared to those who met the Q-LSI of > 90% and controls. The lingual gyrus is active in a number of movement-related processes, fast motor sequences, skill acquisition, movement observation, and muscle contraction steadiness^49–51^. Several reports have also linked lingual gyrus activity in ACL-R patients to greater neural demands for visuo-motor control^19,52^. The relationship between the lingual gyrus and function is largely based on previous neuroimaging investigations which have suggested the lingual gyrus is involved in visual attention and a hub for integrating multiple sensory stimuli^53,54^. In a study investigating brain regions involved in isometric ankle dorsiflexion, Yoon et al.^51^ described the lingual gyrus as a region that integrates visual and attentional stimuli to coordinate appropriate muscle output. Therefore, activity within the lingual gyrus may be critical for coordinated muscle action relative to proprioceptive and visual cues to achieve restored function.

Levels of lingual gyrus activity may also differ based on novelty and motor performance difficulty. For example, Guo et al.^55^ evaluated brain activity between athletes and non-athletes during sport-related visuo-spatial tasks. Interestingly, the “expert” group recruited *less* cortical activity within the bilateral lingual gyrus compared to the non-expert or “novice” group. Thus, during the rehabilitation window, patients with persistent strength asymmetry may develop motor strategies that upweight visuo-motor integration in order to maintain coordination and balance as a consequence of inadequate quadriceps strength. However, it is important to note that our experiment did not explicitly test visuo-motor integration and therefore, the exact function of the lingual gyrus within the context of lower extremity movement as well as muscle asymmetry still needs to be elucidated.

The lingual gyrus and associated extrastriate visual regions identified^19,23^, are also responsible for visual representations of limb and body movements^56,57^. Rather than increases in overt visual input, these regions may be engaged in underlying neural processes of interoceptive awareness. Interoception is defined as the perception of body and limb information^58^. Interestingly, extrastriate visual regions within ACL-R participants have been shown to overlap with neural networks responsible for interception^20,59^. While multidimensional assessments of interoceptive awareness have not been assessed in ACL-R individuals, constructs that overlap with body awareness (e.g. kinesiophobia) have been reported to be psychological factors important for ACL-R recovery^60–63^.

### The premotor cortex and strength asymmetry

We identified cortical activity within the contralateral PMC to be related to strength asymmetry in individuals with a prior history of ACL-R (Fig. 2). While investigations exploring the role of the cortex and muscle activity have been largely focused on the primary motor cortex (M1), the PMC has been shown to influence muscle activity and sensorimotor integration^64^. The PMC receives direct inputs from the dorsolateral prefrontal cortex and the parietal cortex, and projects to the M1 for motor execution. Moreover, the PMC also plays an active role during M1 reorganization after central injury (e.g. ischemic or hemorrhagic stroke)^65^. For example, after an ischemic stroke affecting M1 or the corticospinal tract, the PMC can remain functional and display heightened levels of neuronal activity in concert with increased levels of growth factor release, contributing to recovery^66^. While ACL-R is not a central injury, disruptions in afferent information secondary to joint capsule distension, ligament rupture, and compensatory changes in sensorimotor integration to the affected knee may result in a similar phenomenon, though to a lesser degree^8^.

Considering that neurons within the M1 exhibit greater levels of intracortical inhibition and reductions in excitability in those after ACL injury^15^, increase recruitment of PMC, may, compensate for depressed M1 excitability or excessive inhibition^14,15,67^,This would support conclusions from prior fMRI and electroencephalography investigations, which have collectively provided evidence of increased attention (anterior cingulate region) and motor planning (pre-motor region) associated activity in patients following ACL injury, likely in an attempt to overcome the elevated injury induced inhibtion^19,23,68,69^,Greater PMC activity has also been observed in nonathletes or “novices” compared to athletes or “experts” for voluntary movement control. This work implicates a lack of “neural efficiency” or decreased neural activity to achieve the same behavior in the novices whom require greater levels of attention and thus, neural activity to achieve the same motor performance^70^. Our observations propose a similar manifestation, where those who exhibit greater strength asymmetry may revert *or* adopt a relative “novice” motor brain activation strategy by relying more on pre-motor activity.

### The contralateral superior parietal cortex and strength asymmetry

Despite the a priori ROI selection requiring activity differences in whole brain analyses for the knee motor task between controls and those with ACL-R, the unique role of the parietal cortex in sensory integration for motor control warranted post-hoc investigation^31,32^ (refer to supplemental materials). The contralateral superior parietal cortex correlation with quadriceps strength asymmetry (lower strength in the involved side) may indicate potential sensory compensation to preserve function^71^. The posterior parietal cortex encodes multiple parameters, including movement direction, position, amplitude, velocity and acceleration related to maintaining sensorimotor contorl^72^. A prior cross-sectional investigation with a different knee motor task that also involved hip and ankle flexion identified heightened neural activity within the SPL for individuals after ACL injury relative to healthy controls. In that report, SPL activity was found to be functionally connected to frontal and primary motor activity for lower limb coordination in the ACL-R cohort. Therefore, mechanistic relationships between increased SPL activity in individuals with greater levels of strength asymmetry should be further explored, but may relate to sensory processing requirements cascading into attentional and spatial related processing via frontal-parietal connectivity to compensate for the lack of strength^32^.

### Limitations

The fMRI paradigm in this study to assay neural correlates of knee control was a submaximal quadriceps-dominated task. Consequently, the relationship between brain activity during repeated submaximal movement tasks may differ to a maximal quadriceps contraction. This is especially important when considering that other neurophysiologic measures (e.g. corticospinal excitability) are related to peak torque but not submaximal, coordination tasks in those after ACL-R^12,73^. Consequently, future studies should attempt to investigate neural correlates of maximal quadriceps contractions. While fMRI provides the opportunity to visualize cortical and subcortical neural activity with a high level of spatial and temporal fidelity, head motion, which can be prevalent under high contraction intensities, remains a challenging confound to data quality. Due to MRI technical limitations at the time, we were not able to capture a quantitative measure of task performance, but practice sessions were completed to ensure task timing and all participants were monitored to ensure the same movement rate across participants. The use of more sophisticated fMRI paradigms are becoming more available with methods for quantitative in-scanner biomechanics^74^, and thus, future studies may provide more precise descriptions of neural contributions to knee control.

Further, we quantified strength via Q-LSI. While this is a common metric for measuring levels of strength deficits by clinicians, the contralateral limb has been shown to exhibit weakness following unilateral injury^75^, and Q-LSI can overestimate levels of knee function in patients following ACL-R^76^. As such, future studies may benefit from using normalized measures of strength while incorporating fMRI paradigms that can quantify force-output in the scanner. For reference, average isokinetic strength for ACL-R participants and healthy controls can be found in the supplementary materials.

### Clinical implications perspective

The current investigation found that individuals after ACL-R with underlying strength asymmetry exhibit recruit higher levels of brain activity within regions responsible for visual-spatial integration and motor planning. Therefore, current strategies for ACL-R rehabilitation may not address central adaptations underlying strength asymmetry. Strategies such as cryotherapy and NMES have been suggested as methods to improve deficits in strength^77^. These strategies, which aim to restore neural drive to the muscle via increasing afferent traffic may mitigate strength losses secondary to central nervous system adaptations^77^. These data suggest modifying patient visual attention, clinician instruction, and motor learning principles as a means to modulate neural activity during rehabilitation^78,79^. As selective attention can modulate neural activity within several visuo-spatial and sensorimotor regions (including the PMC and lingual gyrus)^53,80^. Therefore, some of the findings of this investigation may be an indicator of heightened attention in those with greater levels of weakness (increased strength asymmetry). The use of motor learning principles and instructional delivery, which can modulate an individual’s attention during skill acquisition, may mitigate neurophysiologic consequences of injury though further study is needed.

## Conclusion

This study is the first to suggest a link between clinical strength asymmetry and brain activity following ACL-R. ACL-R participants with greater strength asymmetry allocated greater cortical resources for a knee motor task. ACL-R participants, however, who achieve Q-LSI > 90% do not show any cortical activation differences to controls.

## Supplementary Information

Supplementary Information.

## Data Availability

Data is available upon request and institutional review board approval, please contact Dustin Grooms at groomsd@ohio.edu.

## References

[1] Boden BP, Sheehan FT, Torg JS, Hewett TE (2010). Non-contact ACL Injuries: Mechanisms and risk factors. J. Am. Acad Orthop. Surg..

[2] Musahl V, Diermeier T, de Sa D, Karlsson J (2020). ACL surgery: When to do it?. Knee Surg. Sports Traumatol. Arthrosc..

[3] Lepley LK (2015). Deficits in quadriceps strength and patient-oriented outcomes at return to activity after ACL reconstruction: A review of the current literature. Sports Health..

[4] Lewek M, Rudolph K, Axe M, Snyder-Mackler L (2002). The effect of insufficient quadriceps strength on gait after anterior cruciate ligament reconstruction. Clin. Biomech..

[5] Schmitt LC, Paterno MV, Hewett TE (2012). The impact of quadriceps femoris strength asymmetry on functional performance at return to sport following anterior cruciate ligament reconstruction. J. Orthop. Sports Phys. Ther..

[6] Grindem H, Snyder-Mackler L, Moksnes H, Engebretsen L, Risberg MA (2016). Simple decision rules can reduce reinjury risk by 84% after ACL reconstruction: The Delaware-Oslo ACL cohort study. Br. J. Sports Med..

[7] Tourville TW, Jarrell KM, Naud S, Slauterbeck JR, Johnson RJ, Beynnon BD (2014). Relationship between isokinetic strength and tibiofemoral joint space width changes after anterior cruciate ligament reconstruction. Am. J. Sports Med..

[8] Criss CR, Melton MS, Ulloa SA (2021). Rupture, reconstruction, and rehabilitation: A multi-disciplinary review of mechanisms for central nervous system adaptations following anterior cruciate ligament injury. Knee.

[9] Needle AR, Lepley AS, Grooms DR (2017). Central nervous system adaptation after ligamentous injury: A summary of theories, evidence, and clinical interpretation. Sports Med..

[10] Kapreli E, Athanasopoulos S (2006). The anterior cruciate ligament deficiency as a model of brain plasticity. Med. Hypotheses.

[11] Lepley AS, Ericksen HM, Sohn DH, Pietrosimone BG (2014). Contributions of neural excitability and voluntary activation to quadriceps muscle strength following anterior cruciate ligament reconstruction. Knee.

[12] Zarzycki R, Morton SM, Charalambous CC, Pietrosimone B, Williams GN, Snyder-Mackler L (2020). Examination of corticospinal and spinal reflexive excitability during the course of postoperative rehabilitation after anterior cruciate ligament reconstruction. J. Orthop. Sports Phys. Ther..

[13] Zarzycki R, Morton SM, Charalambous CC, Pietrosimone B, Williams GN, Snyder-Mackler L (2020). Athletes after anterior cruciate ligament reconstruction demonstrate asymmetric intracortical facilitation early after surgery. J. Orthop. Res..

[14] Lepley AS, Gribble PA, Thomas AC, Tevald MA, Sohn DH, Pietrosimone BG (2015). Quadriceps neural alterations in anterior cruciate ligament reconstructed patients: A 6-month longitudinal investigation. Scand. J. Med. Sci. Sports..

[15] Luc-Harkey BA, Harkey MS, Pamukoff DN (2017). Greater intracortical inhibition associates with lower quadriceps voluntary activation in individuals with ACL reconstruction. Exp. Brain Res..

[16] Lepley AS, Grooms DR, Burland JP, Davi SM, Kinsella-Shaw JM, Lepley LK (2019). Quadriceps muscle function following anterior cruciate ligament reconstruction: Systemic differences in neural and morphological characteristics. Exp. Brain Res..

[17] Nuccio S, Del Vecchio A, Casolo A (2021). Deficit in knee extension strength following anterior cruciate ligament reconstruction is explained by a reduced neural drive to the vasti muscles. J. Physiol..

[18] Madhavan S, Shields RK (2011). Neuromuscular responses in individuals with anterior cruciate ligament repair. Clin. Neurophysiol..

[19] Grooms DR, Page SJ, Nichols-Larsen DS, Chaudhari AMW, White SE, Onate JA (2017). Neuroplasticity associated with anterior cruciate ligament reconstruction. J. Orthop. Sports Phys. Ther..

[20] Criss CR, Onate JA, Grooms DR (2020). Neural activity for hip-knee control in those with anterior cruciate ligament reconstruction: A task-based functional connectivity analysis. Neurosci. Lett..

[21] Criss CR, Lepley AS, Onate JA (2023). Neural correlates of self-reported knee function in individuals after anterior cruciate ligament reconstruction. Sports Health.

[22] van Melick N, Meddeler BM, Hoogeboom TJ, van der Nijhuis-Sanden MWG, van Cingel REH (2017). How to determine leg dominance: The agreement between self-reported and observed performance in healthy adults. PLoS ONE.

[23] Kapreli E, Athanasopoulos S, Gliatis J (2009). Anterior cruciate ligament deficiency causes brain plasticity: A functional MRI study. Am. J. Sports Med..

[24] Kapreli E, Athanasopoulos S, Papathanasiou M (2006). Lateralization of brain activity during lower limb joints movement An fMRI study. Neuroimage.

[25] Luft AR, Smith GV, Forrester L (2002). Comparing brain activation associated with isolated upper and lower limb movement across corresponding joints. Hum. Brain Mapp..

[26] Luft AR, Forrester L, Macko RF (2005). Brain activation of lower extremity movement in chronically impaired stroke survivors. Neuroimage.

[27] Beckmann CF, Jenkinson M, Smith SM (2003). General multilevel linear modeling for group analysis in FMRI. Neuroimage.

[28] Woolrich MW, Behrens TEJ, Beckmann CF, Jenkinson M, Smith SM (2004). Multilevel linear modelling for FMRI group analysis using Bayesian inference. Neuroimage.

[29] Woolrich M (2008). Robust group analysis using outlier inference. Neuroimage.

[30] Worsley KJ (2001). Statistical analysis of activation images. Funct. MRI: An Introd. Methods..

[31] Fogassi L, Luppino G (2005). Motor functions of the parietal lobe. Curr. Opin. Neurobiol..

[32] Desmurget M, Sirigu A (2009). A parietal-premotor network for movement intention and motor awareness. Trends Cogn. Sci..

[33] Mumford, J. A guide to calculating percent change with featquery. *Unpublished Tech Report In: ht tp://mum fordboluclaedu / perchange_guide pdf*. 2007;177.

[34] Benjamini Y, Hochberg Y (1995). Controlling the false discovery rate: A practical and powerful approach to multiple testing. J. Roy. Stat. Soc.: Ser. B (Methodol.).

[35] Lynch AD, Logerstedt DS, Grindem H (2015). Consensus criteria for defining ‘successful outcome’ after ACL injury and reconstruction: A Delaware-Oslo ACL cohort investigation. Br. J. Sports Med..

[36] Sapega AA (1990). Muscle performance evaluation in orthopaedic practice. J. Bone Joint. Surg. Am..

[37] Adams D, Logerstedt D, Hunter-Giordano A, Axe MJ, Snyder-Mackler L (2012). Current concepts for anterior cruciate ligament reconstruction: A criterion-based rehabilitation progression. J. Orthop. Sports Phys. Ther..

[38] Kvist J (2004). Rehabilitation following anterior cruciate ligament injury: Current recommendations for sports participation. Sports Med..

[39] Cohen J. *Statistical Power Analysis for the Behavioral Sciences*. 2nd edn. Hillsdale, New Jersey: L. Published online (1988).

[40] Stark-Inbar A, Dayan E (2017). Preferential encoding of movement amplitude and speed in the primary motor cortex and cerebellum. Hum. Brain Mapp..

[41] Toxopeus CM, de Jong BM, Valsan G, Conway BA, Leenders KL, Maurits NM (2011). Direction of movement is encoded in the human primary motor cortex. PLoS ONE.

[42] Cramer SC, Weisskoff RM, Schaechter JD (2002). Motor cortex activation is related to force of squeezing. Hum. Brain Mapp..

[43] Dhillon MS, Bali K, Prabhakar S (2012). Differences among mechanoreceptors in healthy and injured anterior cruciate ligaments and their clinical importance. Muscles Ligaments Tendons J..

[44] Rice DA, McNair PJ (2010). Quadriceps arthrogenic muscle inhibition: Neural mechanisms and treatment perspectives. Semin. Arthritis Rheum..

[45] Lepley LK, Davi SM, Burland JP, Lepley AS (2020). Muscle atrophy after ACL injury: Implications for clinical practice. Sports Health.

[46] Neto T, Sayer T, Theisen D, Mierau A (2019). Functional brain plasticity associated with ACL injury: A scoping review of current evidence. Neural Plast..

[47] Rodriguez KM, Palmieri-Smith RM, Krishnan C (2020). How does anterior cruciate ligament reconstruction affect the functioning of the brain and spinal cord? A systematic review with meta-analysis. J. Sport Health Sci..

[48] Tayfur B, Charuphongsa C, Morrissey D, Miller SC (2020). Neuromuscular Function of the knee joint following knee injuries: Does it ever get back to normal? A systematic review with meta-analyses. Sports Med..

[49] Pi YL, Wu XH, Wang FJ (2019). Motor skill learning induces brain network plasticity: A diffusion-tensor imaging study. PLoS ONE.

[50] Decety J, Perani D, Jeannerod M (1994). Mapping motor representations with positron emission tomography. Nature.

[51] Yoon T, Vanden Noven ML, Nielson KA, Hunter SK (2014). Brain areas associated with force steadiness and intensity during isometric ankle dorsiflexion in men and women. Exp. Brain. Res..

[52] Wohl T, Criss CR, Grooms DR (2021). Visual perturbation to enhance return to sport rehabilitation after anterior cruciate ligament injury: A clinical commentary. Int. J. Sports Phys. Ther..

[53] Macaluso E, Frith CD, Driver J (2000). Modulation of human visual cortex by crossmodal spatial attention. Science.

[54] Macaluso E, Driver J (2001). Spatial attention and crossmodal interactions between vision and touch. Neuropsychologia.

[55] Guo Z, Li A, Yu L (2017). “Neural efficiency” of athletes’ brain during visuo-spatial task: An fMRI study on table tennis players. Front. Behav. Neurosci..

[56] Astafiev SV, Stanley CM, Shulman GL, Corbetta M (2004). Extrastriate body area in human occipital cortex responds to the performance of motor actions. Nat. Neurosci..

[57] Amoruso L, Couto B, Ibáñez A (2011). Beyond extrastriate body area (EBA) and fusiform body area (FBA): Context integration in the meaning of actions. Front. Hum. Neurosci..

[58] Herbert BM, Pollatos O (2012). The body in the mind: On the relationship between interoception and embodiment. Top. Cogn. Sci..

[59] Stern ER, Grimaldi SJ, Muratore A (2017). Neural correlates of interoception: Effects of interoceptive focus and relationship to dimensional measures of body awareness. Hum Brain Mapp..

[60] Ardern CL, Taylor NF, Feller JA, Whitehead TS, Webster KE (2013). Psychological responses matter in returning to preinjury level of sport after anterior cruciate ligament reconstruction surgery. Am. J. Sports Med..

[61] Flanigan DC, Everhart JS, Pedroza A, Smith T, Kaeding CC (2013). Fear of Reinjury (Kinesiophobia) and persistent knee symptoms are common factors for lack of return to sport after anterior cruciate ligament reconstruction. Arthros. J. Arthrosc. Relat. Surg..

[62] Tichonova A, Rimdeikienė I, Petruševičienė D, Lendraitienė E (2016). The relationship between pain catastrophizing, kinesiophobia and subjective knee function during rehabilitation following anterior cruciate ligament reconstruction and meniscectomy: A pilot study. Medicina (Kaunas).

[63] Christino MA, Fantry AJ, Vopat BG (2015). Psychological aspects of recovery following anterior cruciate ligament reconstruction. JAAOS–J. Am. Acad. Orthop. Surg..

[64] Fornia L, Rossi M, Rabuffetti M (2020). Direct electrical stimulation of premotor areas: Different effects on hand muscle activity during object manipulation. Cereb. Cortex.

[65] Kantak SS, Stinear JW, Buch ER, Cohen LG (2012). Rewiring the brain: Potential role of the premotor cortex in motor control, learning, and recovery of function following brain injury. Neurorehabil. Neural Repair.

[66] Alia C, Spalletti C, Lai S (2017). Neuroplastic changes following brain ischemia and their contribution to stroke recovery: Novel approaches in neurorehabilitation. Front. Cellular Neurosci..

[67] Dum RP, Strick PL (1991). The origin of corticospinal projections from the premotor areas in the frontal lobe. J. Neurosci..

[68] Baumeister J, Reinecke K, Weiss M (2008). Changed cortical activity after anterior cruciate ligament reconstruction in a joint position paradigm: An EEG study. Scand. J. Med. Sci. Sports.

[69] Baumeister J, Reinecke K, Schubert M, Weiss M (2011). Altered electrocortical brain activity after ACL reconstruction during force control. J. Orthop. Res..

[70] Del Percio C, Babiloni C, Marzano N (2009). “Neural efficiency” of athletes’ brain for upright standing: A high-resolution EEG study. Brain Res. Bull..

[71] Chaput M, Onate JA, Simon JE (2022). Visual cognition associated with knee proprioception, time to stability, and sensory integration neural activity after ACL reconstruction. J. Orthop. Res..

[72] Battaglia-Mayer, A., Caminiti, R. Posterior parietal cortex and arm movement. In: Squire, L. R., ed. *Encyclopedia of Neuroscience*. Academic Press; 2009:783–795. 10.1016/B978-008045046-9.01332-2.

[73] Ward SH, Perraton L, Bennell K, Pietrosimone B, Bryant AL (2019). Deficits in quadriceps force control after anterior cruciate ligament injury: Potential Central mechanisms. J. Athl. Train..

[74] Strong A, Grip H, Boraxbekk CJ, Selling J, Häger CK (2022). Brain Response to a knee proprioception task among persons with anterior cruciate ligament reconstruction and controls. Front. Hum. Neurosci..

[75] Thomas AC, Villwock M, Wojtys EM, Palmieri-Smith RM (2013). Lower extremity muscle strength after anterior cruciate ligament injury and reconstruction. J. Athl. Train..

[76] Wellsandt E, Failla MJ, Snyder-Mackler L (2017). Limb symmetry indexes can overestimate knee function after anterior cruciate ligament injury. J. Orthop. Sports Phys. Ther..

[77] Sonnery-Cottet B, Saithna A, Quelard B (2019). Arthrogenic muscle inhibition after ACL reconstruction: A scoping review of the efficacy of interventions. Br. J. Sports Med..

[78] Gokeler A, Neuhaus D, Benjaminse A, Grooms DR, Baumeister J (2019). Principles of motor learning to support neuroplasticity after ACL injury: Implications for optimizing performance and reducing risk of second ACL injury. Sports Med..

[79] Grooms D, Appelbaum G, Onate J (2015). Neuroplasticity following anterior cruciate ligament injury: A framework for visual-motor training approaches in rehabilitation. J. Orthop. Sports Phys. Ther..

[80] Milnik A, Nowak I, Müller NG (2013). Attention-dependent modulation of neural activity in primary sensorimotor cortex. Brain Behav..

