# Supplementary material for: Brain activity associated with quadriceps strength deficits after anterior cruciate ligament reconstruction
Source: Sci Rep. 2023 May 17;13:8043. doi: 10.1038/s41598-023-34260-2 (PMC10192374; doi:10.1038/s41598-023-34260-2)
Supplement: Supplementary file 1 — Supplementary Information. [file 41598_2023_34260_MOESM1_ESM.docx]

Supplemental materials

| Imaging Parameters | | |
| --- | --- | --- |
|  | Site 1 | Site 2 |
|  | 3.0T Prisma Siemens | 3.0T Magnetom Siemens |
| *Coil* | 20-channel phase array | 12-channel phase array |
| *Functional Imaging* | 2.0 mm^3^ voxel size, 2 mm slice thickness, 90 degree flip angle | 2.5mm^3^ voxel size, 2.5 mm slice thickness, 90 degree flip angle |
| *T1-Weighted Anatomical* | TR: 2530 ms, TE:1.80 ms, field of view:256×256 mm;  A-P phase, slice thickness: 1 mm, 176 slices | TR: 2000 ms; TE: 4.58 ms; field of view: 256×256 mm; A-P  phase; slice thickness:  1 mm; 176 slices |

**Supplemental Table 1**: Imaging parameters for MRI data and scanner descriptions at each testing site.

**MRI paradigm**

Participants were given earphones and a foam roller was placed under their knees. Straps were placed across the participants: one across the thighs at the mid-point between the greater trochanter and knee joint line, one across the hips at the anterior superior iliac spines, and two across the chest from each shoulder, forming an X pattern. To reduce head motion during the functional scans, participants were also fitted using high-density MRI-safe, customized head padding. Participants were fitted with a dorsal night splint (FUTURO™ Night Plantar Fasciitis Sleep Support) to immobilize the ankle. Participants were also monitored during the functional motor run to ensure sufficient range of motion during the motor block, non-compliance, and accessory motions. If participants did not achieve enough range of motion during the task, they were asked to repeat the run.

**First level task image processing**

Imaging data at both scanners underwent separate preprocessing pipelines to reduce inter-scanner variability. All data was preprocessed using software package FSL (FMRIB, Oxford UK) and went as follows: brain extraction, MCFLIRT motion correction, Gaussian kernel FWHM 5mm spatial smoothing spatial smoothing, mean-based intensity normalization of all volumes, and high-pass temporal filtering at 90 Hz.^1–3^ Additionally, due to the nature of the task, all data was also denoised using Independent Component Analysis – based strategy for Automatic Removal of Motion Artifacts (ICA-AROMA) to  reduce confounding signal from head-motion.^4,5^ Anatomical images and standard MNI 152, 2 mm space were co-registered functional scans using linear image registration. For subject-level analysis, FSL FEAT’s General Linear Model was at cluster threshold of z>3.1, p<.05, which are standard imaging parameter thresholds used to reduce false positives.^3,6–9^

**Whole Brain Task Average Results**

The results of the whole brain analysis referenced in the main text for the task average brain activity are reported in supplemental table 2 and supplemental figure 1.

| Cluster Index | Brain Regions | Voxel (#) | p-value | Z-max | MNI coordinate of peak voxel | | | Z center of gravity | | |
| --- | --- | --- | --- | --- | --- | --- | --- | --- | --- | --- |
|  |  |  |  |  | x | y | z | X (mm) | Y (mm) | Z (mm) |
| 3 | Primary motor cortex, Primary sensory cortex, Secondary sensory cortex, Premotor cortex, Corticospinal tract, Superior & inferior parietal lobe | 36288 | <.001 | 9.03 | 2 | -8 | 68 | 1.8 | -21.5 | 42.2 |
| 2 | Lingual gyrus, Occipital fusiform, Cerebellum left I-VI, right I-VI, Vermis | 1402 | <.001 | 6.39 | 2 | -68 | -10 | -1.4 | -54.6 | -10.5 |
| 1 | Frontal pole, Middle frontal gyrus | 175 | 0.016 | 4.15 | 30 | 48 | 24 | 33.4 | 45 | 25.2 |

**Supplemental Table** **2**. The results of the whole brain average analysis reported by cluster findings. P-values are gaussian random field cluster corrected at 3.1, p<.05 at the subject level and group level with FMRIB’s Local Analysis of Mixed Effects (FLAME) stage 1 and stage 2.^3,10,11^


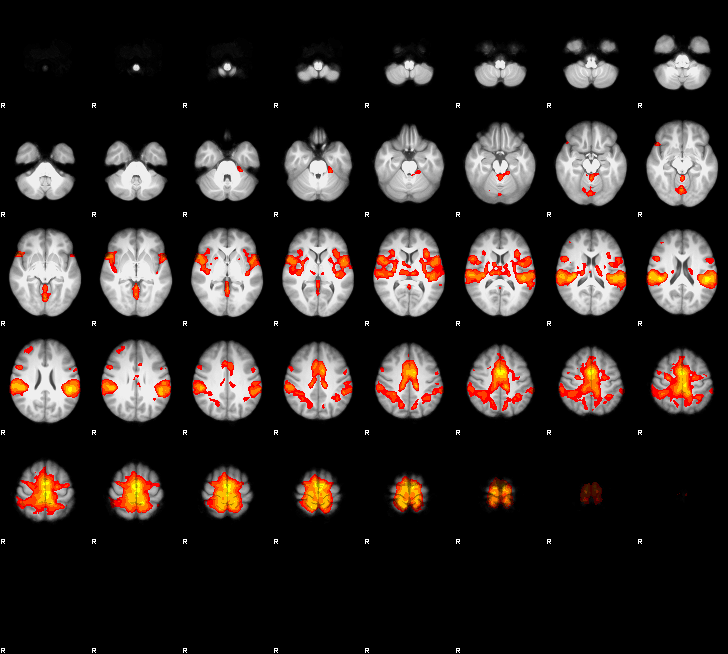


3.1 
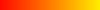
 9.0

**Supplementary Figure 1**. Visual of the whole brain group level average for the knee motor task.

**Exploratory Parietal Cortex Analyses and Result**

The parietal cortex is highly involved in sensory integration and might have a unique relationship to strength loss and associated compensations after ACL-R. Therefore, the contralateral and ipsilateral superior and inferior parietal cortices were included as post-hoc exploratory results. The ROI processing of including only task active voxels within the anatomical region (atlas threshold >30%) was the same for the parietal cortex as those in the main text. These parietal cortex ROIs are considered exploratory as prior comparisons between ACL-R and control cohorts for whole brain activation group differences have not found a difference in these regions. However, the results indicate greater contralateral superior parietal cortex is correlated to greater strength loss of the involved quadriceps or a lower LSI (i.e., increased asymmetry) in the ACL-R group and not in the control group. Although this region is not originally part of our *a priori* hypothesis, we included these additional exploratory analyses below and discussed the unique relationship within the main text (**Supplementary Table 3, 4 and Figure 2 & 3**).

| ROI | Q-LSI | Q-LSI  **covariate: sex** |
| --- | --- | --- |
| **Ipsilateral Superior Parietal Cortex** | -.228 (.308) | -.231(.314) |
| **Contralateral Superior Parietal Cortex** | **-.530(.011)** | **-.552 (.009)** |
| **Ipsilateral Inferior Parietal Cortex** | -.334 (.129) | -.341(.131) |
| **Contralateral Inferior Parietal Cortex** | .051(.820) | .054(.815) |

**Supplementary Table 3.** Relationship between Q-LSI (quadriceps limb symmetry index) and parietal cortex activity of the ACL-R (anterior cruciate ligament reconstruction) cohort.

| ROI | Q-LSI | | Q-LSI  **covariate: sex** |
| --- | --- | --- | --- |
| **Ipsilateral Superior Parietal Cortex** | -.002 (.994) | | -.015(.948) |
| **Contralateral Superior Parietal Cortex** | -.124(.583) | -.135 (.561) | |
| **Ipsilateral Inferior Parietal Cortex** | -.122 (.587) | | -.134(.564) |
| **Contralateral Inferior Parietal Cortex** | -.138(.540) | | -.144(.534) |

**Supplementary Table 4**. Relationship between Q-LSI (quadriceps limb symmetry index) and parietal cortex activity of the healthy controls.

**Supplementary Figure 2:** Bivariate scatterplot of % signal change of the contralateral superior parietal lobule and Q-LSI (quadriceps limb symmetry index) for the ACL-R cohort.

**Supplementary Figure 3.** Group comparison of estimated marginal means of regional % signal change for parietal cortex ROIs related to strength asymmetry (covariate: sex). Error bars, standard error of the mean. Q-LSI < 90%: 12 ACL-R participants with Q-LSI < 90%; 10 ACL-R participants with Q-LSI ≥ 90%; Controls: 22 healthy participants. No significant differences. Q-LSI (quadriceps limb symmetry index).

**Primary Analyses Controlling for Site**

To evaluate the effects of study site (MRI location) in this two-center study, we also completed an analysis of the primary study correlations between region of interest brain activity and quadriceps limb symmetry index (**Supplementary Table 5**). There was no difference in regions identified to correlate with quadriceps limb symmetry when controlling for MRI site.

| ROI | Q-LSI  **covariate: MRI Site (Site 1 n=9; Site 2 n=13)** |
| --- | --- |
| **Ipsilateral M1** | -.368(.10) |
| **Contralateral M1** | -.198 (.39) |
| **Ipsilateral S1** | -.284(.21) |
| **Contralateral S1** | -.354(.16) |
| **Ipsilateral PMC** | -.090 (.70) |
| **Contralateral PMC** | **-.510 (.02)** |
| **Ipsilateral SMA** | -.135 (.56) |
| **Contralateral SMA** | -.115 (.62) |
| **Ipsilateral SII** | -.443 (.04) |
| **Contralateral SII** | -.157 (.497) |
| **Ipsilateral Cerebellum** | -.283(.214) |
| **Contralateral Cerebellum** | -401(.07) |
| **Lingual Gyrus** | **-.562 (.01)** |
| **Ipsilateral Superior Parietal Cortex** | -.210(.36) |
| **Contralateral Superior Parietal Cortex** | **-.563(.01)** |
| **Ipsilateral Inferior Parietal Cortex** | -.373(.10) |
| **Contralateral Inferior Parietal Cortex** | .094(.68) |

**Supplementary Table 5**. Relationship between Q-LSI (quadriceps limb symmetry index) and region of interest brain activity of the ACL-R (anterior cruciate ligament reconstruction) cohort controlling for MRI site.

**References**

1. Jenkinson M, Bannister P, Brady M, Smith S. Improved optimization for the robust and accurate linear registration and motion correction of brain images. *Neuroimage*. 2002;17(2):825-841. doi:10.1016/s1053-8119(02)91132-8

2. Smith SM. Fast robust automated brain extraction. *Hum Brain Mapp*. 2002;17(3):143-155. doi:10.1002/hbm.10062

3. Woolrich MW, Ripley BD, Brady M, Smith SM. Temporal Autocorrelation in Univariate Linear Modeling of FMRI Data. *NeuroImage*. 2001;14(6):1370-1386. doi:10.1006/nimg.2001.0931

4. Pruim RHR, Mennes M, van Rooij D, Llera A, Buitelaar JK, Beckmann CF. ICA-AROMA: A robust ICA-based strategy for removing motion artifacts from fMRI data. *Neuroimage*. 2015;112:267-277. doi:10.1016/j.neuroimage.2015.02.064

5. Pruim RHR, Mennes M, Buitelaar JK, Beckmann CF. Evaluation of ICA-AROMA and alternative strategies for motion artifact removal in resting state fMRI. *Neuroimage*. 2015;112:278-287. doi:10.1016/j.neuroimage.2015.02.063

6. Smith SM, Jenkinson M, Woolrich MW, et al. Advances in functional and structural MR image analysis and implementation as FSL. *Neuroimage*. 2004;23 Suppl 1:S208-219. doi:10.1016/j.neuroimage.2004.07.051

7. Woolrich MW, Behrens TEJ, Smith SM. Constrained linear basis sets for HRF modelling using Variational Bayes. *NeuroImage*. 2004;21(4):1748-1761. doi:10.1016/j.neuroimage.2003.12.024

8. Woolrich MW, Behrens TEJ, Beckmann CF, Jenkinson M, Smith SM. Multilevel linear modelling for FMRI group analysis using Bayesian inference. *Neuroimage*. 2004;21(4):1732-1747. doi:10.1016/j.neuroimage.2003.12.023

9. Eklund A, Nichols TE, Knutsson H. Cluster failure: Why fMRI inferences for spatial extent have inflated false-positive rates. *Proc Natl Acad Sci USA*. 2016;113(28):7900-7905. doi:10.1073/pnas.1602413113

10. Beckmann CF, Jenkinson M, Smith SM. General multilevel linear modeling for group analysis in FMRI. *Neuroimage*. 2003;20(2):1052-1063.

11. Woolrich M. Robust group analysis using outlier inference. *Neuroimage*. 2008;41(2):286-301. doi:10.1016/j.neuroimage.2008.02.042
